# Prise en charge thérapeutique des paragangliomes malins: revue de littérature à travers un cas illustratif

**DOI:** 10.11604/pamj.2019.32.62.8812

**Published:** 2019-02-05

**Authors:** Leila Afani, Hassan Errihani, Ahmad Awada

**Affiliations:** 1 Service d’Oncologie Médicale, Institut National d’Oncologie, Rabat, Maroc; 2 Service d’Oncologie Médicale, Institut Jules Bordet, Bd de Waterloo, Bruxelles, Belgique

**Keywords:** Paragangliome malin, chirurgie, radiothérapie interne, chimiothérapie, Malignant paraganglioma, surgery, internal radiotherapy chemotherapy

## Abstract

Les paragangliomes malins présentent un réel challenge pour les praticiens. Il s'agit de tumeurs rares, de caractérisation complexe, et très hétérogènes dans leur évolution et pronostic. Devant la rareté de ce groupe tumoral, il n'existe pas de consensus sur la prise en charge thérapeutique. A travers ce cas illustratif, nous rapportons l'observation d'une patiente de 29 ans suivie pour un paragangliome malin traitée initialement par chirurgie. Après six mois d'intervalle, récidive multifocale fixant à l'Octreo PET-CT (Positron Emission Tomography- Computer Tomography), la patiente a été mise sous injection mensuelle de Somatuline pendant un an puis arrêté suite à une progression. Une 2^ème^ chirurgie de cytoréduction a été réalisée suivie d'une radiothérapie. Après un an, la patiente a présenté une progression massive. Une chimiothérapie à base de Dacarbazine a été initiée et dont l'évaluation a montré une réponse métabolique quasi complète après huit cures. Le but de ce travail est d'exposer les différentes options thérapeutiques possibles dans la prise en charge des paragangliomes malins.

## Abstract (en)

Malignant paragangliomas pose a real challenge for the practitioners. They are rare complex tumors, very heterogeneous in their evolution and prognosis. Given the rarity of this tumor group, there is no consensus on therapeutic management. Through this illustrative case study, we report the case of a 29 year old female patient followed for malignant paraganglioma due to which she initially had surgery. After a six month interval, multifocal recurrence was detected on Octreoscan PET-CT (Positron Emission Tomography- Computer Tomography). The patient underwent monthly injection of Somatuline for one year and then discontinued therapy due to disease progression. Cytoreduction was then performed followed by radiotherapy. After a year, the patient had massive disease progression. Dacarbazine-based chemotherapy was initiated. The patient had an almost complete metabolic response after eight cycles. This study aims to highlight the different therapeutic options in the management of malignant paragangliomas.

## Introduction

Les paragangliomes abdominaux sont des tumeurs neuroendocrines de localisation extra-surrénalienne. Ils se développent au dépend du système nerveux sympathique. Ce sont des tumeurs rares avec une incidence annuelle de 1/300000 [1]. Il s'agit de tumeurs bénignes, seules 10 à 15% sont malignes [2]. La prise en charge des paragangliomes malins nécessite une collaboration multidisciplinaire. A travers ce cas clinique, nous proposons une revue de littérature de cette entité rare avec discussion des différentes options thérapeutiques en situation métastatique.

## Patient et observation

**Figure 1:**
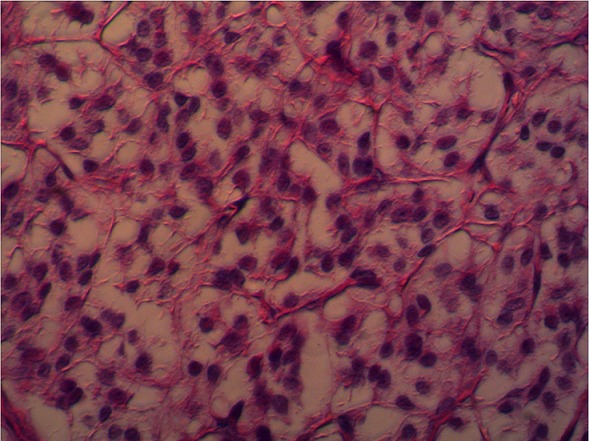
Aspect microscopique du paragangliome montrant des amas arrondis de cellules cuboïdes séparés par des septas fibreux richement vascularisés

Il s'agit d'une patiente de 29 ans d'origine camerounaise sans antécédents notables. En mai 2011, la patiente consulte aux urgences dans un tableau de douleurs abdominales, vomissements et céphalées. L'examen clinique a retrouvé une patiente tachycardique avec un pic hypertensif à 200mmHg. L'examen de l'abdomen n'objectivait pas de masse, il était souple et indolore et les aires ganglionnaires étaient libres. Le reste de l'examen somatique était sans particularités. La chromagranine était à 52,3U/L et le NSE à 16,2ng/ml. Les catécholamines urinaires étaient positives avec la noradrenaline urinaire qui était à 399ug/24h et la normetanephrine à 4432ug/24h. Une IRM abdominale montrait un conglomérat ganglionnaire avec effet de masse sur l'axe iliaque primitif gauche qui fixait à la scintigraphie à la MIBG- I131. Lors d'une laparotomie exploratrice, la patiente a bénéficié d'une exérèse d'une masse de 12x10cm de l'organe de zuckerkandl au niveau de la bifurcation de l'aorte. L'examen anatomopathologique était en faveur d'un paragangliomme malin (Figure 1). Une étude génétique à la recherche en particulier d'une mutation du gène SDHB a été réalisée. Aucune mutation, délétion ou duplication n'a été mise en évidence excluant une cause héréditaire. Après six mois, récidive de la symptomatologie.

L'Octreo PET-CT a montré une récidive multifocale au niveau abdominal montrant une expression neuroendocrine très intense. Fin Novembre 2011, la patiente a été mise sous injection mensuelle de somatuline à la dose de 120mg. En septembre 2012, suite à l'augmentation de la chromagranine, Le bilan radiologique a objectivé une deuxième récidive du paragangliome avec extension ganglionnaire au niveau para-aortique. Après concertation multidisciplinaire, une chirurgie a été indiquée. En avril 2013, l'exploration chirurgicale retrouvait une masse pré-aortique et latéro-aortique avec accolement au grêle majeur dont une boucle contient une masse endoluminale et une masse parasigmoidienne: une réduction maximale a été réalisée. L'étude histologique était en faveur de métastases ganglionnaires et péritonéales du paragangliome malin connu. Devant l'absence de captation à la scintigraphie à la MIBG-I131, une radiothérapie fractionnée à l'IMRT et IGRT a été initiée en août et terminée en octobre 2013. En juillet 2014, nouvelle progression de la maladie. Le PET-FDG montrait une hyperfixation intense au niveau des adénopathies sus-claviculaires gauches, para-aortiques gauches, rétro-péritonéales, iliaques primitives, iliaques internes et externes, péritonéales, para-vésicales et hépatiques. La patiente a été mise sous Dacarbazine à la dose de 1000mg/m2 toutes les trois semaines. L'évaluation post 8 cures a objectivé une réponse quasi complète au PET- FDG 9. La patiente est depuis sous surveillance.

## Discussion

Les paragangliomes sont définis comme des tumeurs neuroendocrines vasculaires rares du paraganglion qui dérivent de la crête neurale. Elles peuvent prendre origine du tissu sympathique surrénalien (phéochromocytome) et extra surrénalien (paragangliome sympathique) ou du tissu parasympathique de la tête et cou [3]. Les paragangliomes sympathiques extra surrénaliens sont rares et représentent 10% de l'ensemble des paragangliomes. Ils se situent au niveau du médiastin et au niveau des chaines ganglionnaires sympathiques para-aortiques lombaires et pelviennes incluant l'organe de zuckerkandl. Ils peuvent survenir à tout âge avec un pic d'incidence entre 40 et 50 ans [4]. Les paragangliomes peuvent être sporadiques ou associés à un syndrome héréditaire: maladie de Von-Hippel-Lindau, la néoplasie endocrinienne multiple type 2, la neurofibromatose type 1 héréditaire et récemment une mutation au niveau de l'une des quatre sous unités du gène SDH [5]. 10 à 15% des paragangliomes sont asymptomatiques. La symptomatologie est liée à la sécrétion des catécholamines avec comme tableau typique une hypertension artérielle paroxystique, des céphalées, des palpitations et des sueurs. Le dosage de la métanephrine libre plasmatique ou la métanephrine urinaire fractionnée présente une sensibilité supérieure à 96%. La chromagranine est souvent élevée même dans les paragangliomes non sécrétants. Son taux est corrélé à la masse tumorale et peut être ainsi un marqueur utile [6]. L'imagerie par TDM et IRM permet de localiser et stadifier la maladie mais avec une faible spécificité. La scintigraphie au MIBG marqué à l'iode 123 ou 131 a une sensibilité et une spécificité élevée dans les tumeurs primitives et très faible en situation métastatique. Le TEP à la F-DOPA présente une meilleure sensibilité diagnostique que la scintigraphie au MIBG. En situation métastatique, le TEP à la FDG est recommandé avec une sensibilité supérieure dans les paragangliomes métastatiques avec mutation SDHB [7].

Les paragangliomes malins représentent 10 à 15%. Ils sont définis par la présence de métastases au niveau de sites dépourvus de chromaffine [2]. Les paragangliomes malins sont souvent associés à la mutation SDHB [7]. Devant la rareté de ces tumeurs, il n'existe pas de stratégie thérapeutique codifiée. La prise en charge doit être dans un cadre multidisciplinaire. Devant l'absence de traitement curatif et la présence de longs survivants, la surveillance peut être une option thérapeutique. Le traitement se justifie devant la présence d'un volume tumoral important, la présence d'un syndrome hormonal incontrôlable et une progression radiologique selon le critère Recist [8]. La chirurgie de cytoréduction permettrait de réduire les symptômes et améliorer la réponse aux autres traitements [9]. Le traitement par iode 131 MIBG chez les patients avec une scintigraphie MIBG positive permettrait un taux de réponse objective de 30% et une stabilité de la maladie dans 57% des cas [10]. Le traitement par les analogues de la somatostatine comme le Yttirium-90-DOTA-TOC et le Lutetium-177-DOTATATE a montré des taux de réponse modeste [11]. La radiothérapie externe seule ou associée à un traitement par Iode 131-MIBG peut être une option thérapeutique dans le contrôle local de la maladie [12]. En absence d'études prospectives, le traitement systémique optimal reste à définir. Le protocole le plus utilisé combinant Dacarbazine, Vincristine et Cyclophosphamide (CVD) est associé à un taux de réponse partielle de 37% [13]. Notre patiente a présenté une réponse métabolique complète après une monochimiothérapie à base de Dacarbazine. Le temolozomide donnait des taux de réponse similaire au CVD particulièrement dans les mutations SDHB et avec un meilleur profil de toxicité [14]. Le sunitinib a été testé chez 17 patients avec 3 réponses partielles et 5 stabilités [15]. Autres thérapies ciblées ont été testées tel que l'everolimus et l'imatinib mais sans réponse objective démontrée [16, 17]. Malgré la présence de longs survivants, le pronostic de ces tumeurs reste sombre avec un taux de survie globale à 5ans de 20 à 50% [2].

## Conclusion

Les paragangliomes malins sont des tumeurs rares et agressives. Ce cas clinique illustre les différentes options thérapeutiques possibles. Cependant, leur prise en charge reste très complexe et spécifique. Des essais prospectifs sont nécessaires pour une meilleure caractérisation de ces tumeurs afin de pouvoir identifier le traitement adéquat.

## Conflits d’intérêts

Les auteurs ne déclarent aucun conflit d'intérêts.
